# Matrix Metalloproteinases: The Gene Expression Signatures of Head and Neck Cancer Progression

**DOI:** 10.3390/cancers6010396

**Published:** 2014-02-13

**Authors:** Shinji Iizuka, Naozumi Ishimaru, Yasusei Kudo

**Affiliations:** 1 Sanford-Burnham Medical Research Institute, 10901 North Torrey Pines Road, La Jolla, CA 92037, USA; E-Mail: siizuka@sanfordburnham.org; 2 Department of Oral Molecular Pathology, Institute of Health Biosciences, The University of Tokushima Graduate School, 3-8-15 Kuramoto, Tokushima 770-8504, Japan; E-Mail: ishimaru.n@tokushima-u.ac.jp

**Keywords:** matrix metalloproteinase, invasion, angiogenesis, head and neck squamous cell carcinoma

## Abstract

Extracellular matrix degradation by matrix metalloproteinases (MMPs) plays a pivotal role in cancer progression by promoting motility, invasion and angiogenesis. Studies have shown that MMP expression is increased in head and neck squamous cell carcinomas (HNSCCs), one of the most common cancers in the world, and contributes to poor outcome. In this review, we examine the expression pattern of MMPs in HNSCC by microarray datasets and summarize the current knowledge of MMPs, specifically MMP-1, -3, -7 -10, -12, -13, 14 and -19, that are highly expressed in HNSCCs and involved cancer invasion and angiogenesis.

## 1. Introduction

Cancer is a major disease and cause of death in the world. Over 1.5 million people in the United States were diagnosed with cancer in 2012, and 2.5% of all cancer cases were head and neck squamous cell carcinomas (HNSCCs) [1,2]. Metastatic dissemination of HNSCCs initially occurs to the regional lymph nodes in the neck, and high mortality and poor prognosis of HNSCCs are predicted by occurrence of lymph node metastasis [3]. In some tumor types including HNSCCs, local invasion of the tumor is rarely controlled by surgery alone. There is a pressing need for new therapeutic interventions that target invasive and metastatic HNSCCs.

The extracellular matrix (ECM) undergoes significant remodeling during tumor progression, which is mediated largely by extracellular proteinases, particularly the matrix metalloproteinases (MMPs) [4]. MMPs represent a family of zinc-dependent proteinases, which degrade ECM components, such as collagens and proteoglycans, and they have a role not only in normal development and tissue damage in various pathophysiological conditions such as arthritis and wound healing but also in tumor development [5]. MMPs have been implicated in the promotion of tumor invasion and metastasis for decades [6]. More than 20 different human MMPs have been identified [7], and many MMPs are expressed and contribute to HNSCC progression [8,9].

## 2. MMP Expression in HNSCCs

**Figure 1:**
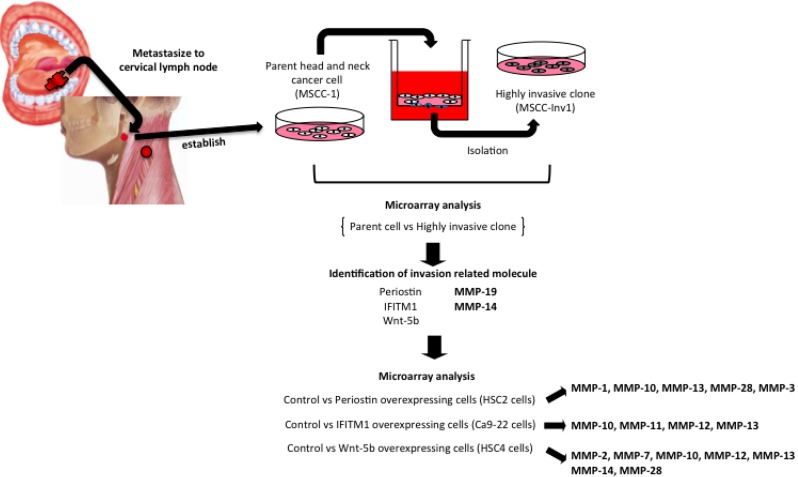
Up-regulated MMP genes in highly invasive HNSCC cell lines as compared to parental cell lines. Adapted from ref. [10] and Table 1 with modification.

Widespread use of gene expression microarrays has identified potential candidate genes in HNSCCs. Further investigation of the biological behaviors and significant clinical outcome of these genes in HNSCCs is of great interest. We previously established a HNSCC cell line, MSCC-1, from lymph node metastasis, and then we isolated a highly invasive clone, MSCC-Inv1, from MSCC-1 using an *in vitro* invasion assay device (Figure 1) [4]. Gene expression profiles of both MSCC-1 and MSCC-Inv1 were compared by microarray analysis, and invasion-related molecules, such as periostin (originally named osteoblast-specific factor-2), interferon-induced transmembrane protein 1 (IFITM1) and Wnt-5b were identified (Figure 1) [10,11,12]. From these results, MMP-19 and membrane type 1-MMP (MT1-MMP) were also identified as cancer invasion-related factors (Figure 1 and Table 1). Interestingly, the highly invasive cell lines with overexpression of these three genes (periostin, IFITM1 and Wnt-5b) commonly induced MMP-10 and MMP-13 expression (Figure 1 and Table 1). Pyeon *et al*. have analyzed genome-wide expression profiles of 84 HNSCCs, cervical cancers and site-matched normal epithelial samples that were laser-capture-microdissected from adjacent sections. Comparison of HNSCCs and normal tissues indicated that expression of MMP-1, -3, -7, -9, -10, -12 and -13 was up-regulated in HNSCC cases (Table 2) [13]. In addition, Stokes *et al*. have reported the expression profiles of degradome components in the tumor microenvironment of HNSCCs. In this report, comprehensive expression profiling of MMPs was obtained using quantitative real-time reverse transcription-PCR analysis of tissue samples representing the tumor (83 cases), the invasive margin (41 cases) and adjacent tissue (41 cases) from HNSCC patients, along with normal tissue controls (13 cases). The results show that majority of the MMPs were expressed in tumor tissues, both in the center and the invasive margin of the tumor, and that the expression was generally increased as compared to the normal tissue (Table 3) [14]. Furthermore, Ye *et al*. have demonstrated that MMP-1, -3, -7, -9, -10, -11, -12 and -13 were up-regulated in oral tongue squamous cell carcinoma cases (53 primary and 22 matching normal tissues) by genome-wide transcriptomic profiles (Table 4) [15].

**Table 1:** Up-regulated MMP genes in highly invasive HNSCC cell lines as compared to parental cell lines. Adapted from ref. [10] with modification. Cutoff: Fold increased >2.0.

Comparison (/Parent Cells)	Fold Change	Symbol	Other Designations
MSCC-Inv1	2.82	MMP-19	MMP-18, RASI-1
	2.44	MMP-14	MT1-MMP
Periostin overexpressing cells	30.2	MMP-1	Interstitial collagenase
	17.4	MMP-10	Stromelysin 2
	14.7	MMP-13	Collagenase 3
	2.9	MMP-28	Epilysin
	2.6	MMP-3	Stromelysin 1, Progelatinase
IFITM1 overexpressing cells	19.1	MMP-13	Collagenase 3
	8.2	MM-12	Macrophage elastase
	3.6	MMP-10	Stromelysin 2
	2.8	MMP-11	Stromelysin 3
Wnt-5B overexpressing cells	7.9	MMP-12	Macrophage elastase
	7.8	MMP-13	Collagenase 3
	6.1	MMP-7	Matrilysin, uterine
	5.8	MMP-10	Stromelysin 2
	2.8	MMP-14	MT1-MMP
	2.5	MMP-28	Epilysin
	2.2	MMP-2	72 kDa gelatinase, type IV collagenase

**Table 2:** Up-regulated MMP genes in HNSCC cases as compared to normal tissues. Adapted from ref. [13] with modification. Cutoff: Fold increased >2.0.

Fold Change	Symbol	Other Designations
13.9	MMP-1	Interstitial collagenase
12.0	MMP-12	Macrophage elastase
8.6	MMP-10	Stromelysin 2
8.1	MMP-3	Stromelysin 1
7.3	MMP-7	Matrilysin, uterine
7.2	MMP-9	92kDa gelatinase, type IV Collagenase
5.7	MMP-13	Collagenase 3

* *p* value < 0.01; fold increase >2.0.

**Table 3:** Up-regulated MMP genes in HNSCC cases as compared to normal tissues. Adapted from ref. [14] with modification.

Symbol	Other Designations	*p* value
MMP-1	Interstitial collaganase	**
MMP-2	72 kDa gelatinase, type IV collagenase	**
MMP-3	Stromelysin 1	**
MMP-7	Matrilysin, uterine	**
MMP-8	PMNL collagenase (MNL-CL)	*
MMP-9	92 kDa gelatinase, type IV Collagenase	**
MMP-10	Stromelysin 2	**
MMP-11	Stromelysin 3	**
MMP-12	Macrophage elastase	**
MMP-13	Collagenase 3	**
MMP-14	MT1-MMP	**
MMP-15	MT2-MMP	NS
MMP-16	MT3-MMP	**
MMP-17	MT4-MMP	**
MMP-19	MMP-18, RASI-1	**
MMP-23	CA-MMP	**
MMP-24	MT5-MMP	**
MMP-25	MT6-MMP	**
MMP-27	MMP-27	NS
MMP-28	Epilysin	NS

*p* value: *, *p* = 0.01–0.05; **, *p* < 0.001; NS, not significant.

**Figure 2:**
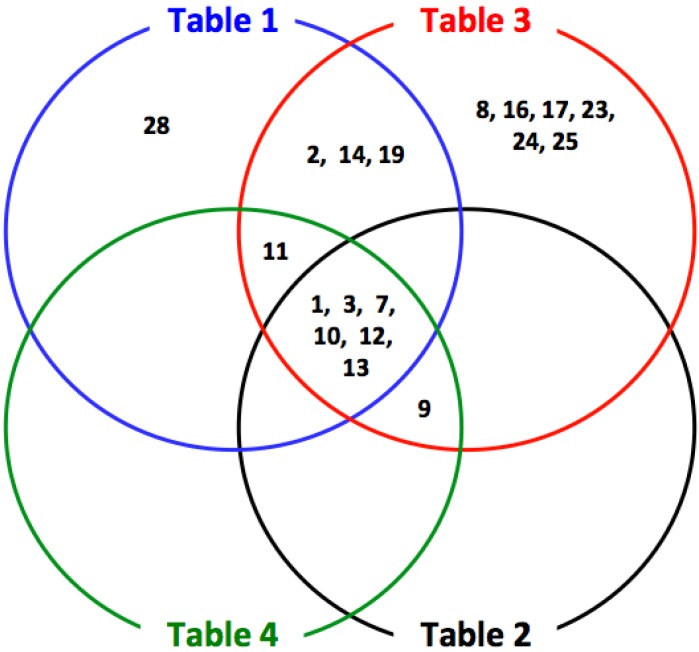
Venn diagram depicting the extent of overlap between the MMP expression profiles from Table 1, Table 2, Table 3 and Table 4.

**Figure 3:**
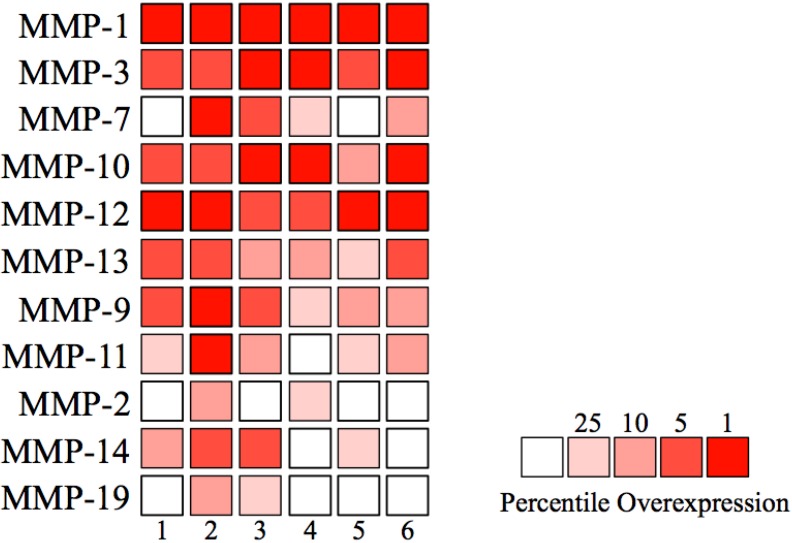
The heat map of MMP expression in HNSCC. Each number of analyses is corresponding to Table 3. The heat map intensity corresponds to percentile overexpression (red).

Taken together with results of MMP gene expression profiling in HNSCCs (Table 1, Table 2, Table 3 and Table 4), MMP-1, -3, -7, -10, -12 and -13 may strongly correlate with HNSCC tumorigenicity and progression (Figure 2). To confirm the MMP expression pattern in Figure 2, 11 MMPs (MMP-1, -2, -3, -7, -9, -10, -11, -12, -13, -14 and -19) that increased in at least two independent gene expression profiling datasets were examined by the Oncomine public cancer microarray database [16]. As expected, MMP-1, -3, -7, -10, -12 and -13 (identified as the most highly expressed MMPs in HNSCC by four independent gene expression datasets in Figure 2) were significantly increased in almost all HNSCC microarray datasets (Table 5 and Figure 3). In Table 5, MMP-1, -3, -7, -9, -10, -11, -12 and -13 demonstrate strong significance and correlation between the six cited studies, MMP-2, -14 and -19 did not. This may be attributed to the fact that RNA samples used in each profiling datasets were obtained from different stage of HNSCC. Therefore, we think that MMP-2, -14 and -19 were not up-regulated in some datasets. In fact, our previous study showed that MMP-14 expression is significantly increased in highly invasive cell line, MSCC-inv1 cells compare to parent cells. These findings suggest that some of MMP expression may be locally regulated in part of tumor tissue.

**Table 4:** Up-regulated MMP genes in HNSCC cases as compared to normal tissues. Adapted from ref. [15] with modification. Cutoff: Fold increased >2.0.

Fold Change	Symbol	Other Designations
57.6	MMP-1	Interstitial collaganase
8.4	MMP-10	Stromelysin 2
8.4	MMP-3	Stromelysin 1
7.8	MMP-12	Macrophage elastase
4.1	MMP-9	92 kDa gelatinase, type IV Collagenase
3.8	MMP-13	Collagenase 3
2.8	MMP-7	Matrilysin, uterine
2.0	MMP-11	Stromelysin 3

In this review, we will summarize the role of these MMPs that are highly expressed in HNSCCs, especially MMP-1, -3, -7, -9, -10, -12, -13 and -19, and also review the roles of MMP-14, which is one of the key molecules involved in HNSCC progression through invadopodia formation. 

**Table 5:** MMP expression in HNSCCs using the Oncomine gene expression database. Human HNSCC samples (n = 201) compared against normal tissues (n = 109) from six independent microarray datasets for MMP expression using the Oncomine database. These six datasets (including two microarray datasets from Table 2 and Table 4) were chosen because all other datasets analyzed by less than 10 samples both in normal and in HNSCC were removed. * *p* < 0.05.

Number	Author	Number of samples		MMP-1		MMP-3		MMP-7		MMP-10		MMP-12	
		Normal	**HNSCC**	Fold Change	*p* value	Fold Change	*p* value	Fold Change	*p* value	Fold Change	*p* value	Fold Change	*p* value
1	**Estilo**	26	31	61.7	*	14.6	*	3.0	*	23.6	*	5.8	*
2	**Ginos**	13	41	221.8	*	11.9	*	7.5	*	14.9	*	15.6	*
3	**Peng**	22	57	86.3	*	29.9	*	4.8	*	25.6	*	17.3	*
4	**Pyeon**	10	15	137.5	*	19.0	*	3.8	*	24.1	*	27.6	*
5	**Talbot**	26	31	27.7	*	3.5	*	1.5	0.066	4.0	*	4.1	*
6	**Ye**	12	26	124.6	*	10.6	*	2.2	*	6.4	*	7.3	*
Total		109	201										
Number	Author	MMP-13		MMP-9		MMP-11		MMP-2		MMP-14		MMP-19	
		**Fold Change**	***P* value**	**Fold Change**	***p* value**	**Fold Change**	***p* value**	**Fold Change**	***p* value**	**Fold Change**	***p* value**	**Fold Change**	***p* value**
1	**Estilo**	20.3	*	9.5	*	2.3	*	1.1	0.356	1.9	*	-1.2	0.953
2	**Ginos**	15.2	*	11.8	*	5.3	*	2.2	*	1.8	*	8.8	*
3	**Peng**	9.2	*	3.8	*	2.3	*	1.4	*	2.1	*	1.2	*
4	**Pyeon**	4.3	*	8.1	*	1.4	*	1.8	*	1.0	0.257	1.1	0.286
5	**Talbot**	4.7	*	3.2	*	1.6	*	1.0	0.439	1.1	*	-1.2	1
6	**Ye**	4.0	*	4.6	*	1.5	*	1.1	0.055	1.1	0.159	1.1	0.201

## 3. Roles of MMPs in HNSCCs

### 3.1. MMP-1 in HNSCCs

MMP-1, interstitial collagenase, degrades components of the ECM, such as many types of collagens and proteoglycans. Expression of MMP-1 has been observed in various cancers such as breast, colorectal, gastric and esophageal cancer, and contributes to cancer progression [17,18,19,20,21,22,23]. Animal models using tissue-specific overexpression of MMP-1 in the suprabasal layer of skin have indicated that MMP-1 induced epidermal hyperplasia and increased susceptibility to tumorigenesis [24]. Moreover, MMP-1 in the stromal-tumor microenvironment promotes cancer invasion and angiogenesis of breast cancers through proteolytic cleavage of PAR-1 (protease-activated receptors), which is one of the proteolytically activated G-protein coupled receptors [25,26]. Recent experimental data also show that elevated expression of MMP-1 promotes not only breast cancer growth at the primary site (orthotopic injection model) but also brain and lung metastases (left ventricle of the heart and tail vein injection model) through activation of epidermal growth factor receptor (EGFR) signaling by MMP-1-proteolyzed-active TGFα [27].

Gene profiling datasets showed that MMP-1 expression was strongly increased in HNSCC cases (e.g., 30.2-fold changes in a highly invasive HNSCC cell line, 13.9-fold changes in HNSCC cases and 57.6-fold changes in tongue squamous cell carcinoma cases (Table 1, Table 2 and Table 4, respectively)) [13,14,15]. In addition, other reports have also suggested that MMP-1 expression is up-regulated in HNSCCs [8,28,29,30,31]. Moreover, MMP-1 expression is down-regulated by the tumor-suppressor gene p53 via the transactivator AP-1 [32,33,34]. p53 is one of the most important molecules in HNSCC cancer tumorigenicity, because somatic mutations in the p53 gene, *TP53*, have been found in 60%–80% of HNSCC cases and were associated with a poor outcome of HNSCCs [35,36]. These findings also demonstrate high expression levels of MMP-1 in HNSCCs. On the other hand, a more recent report indicates that elevation of p53 protein levels by cisplatin or 5-FU was markedly reduced by overexpression of periostin in gastric cancer cells [10]. This result suggests that periostin-mediated inhibition of p53 expression may regulate MMP-1 expression in HNSCCs. In fact, our previous microarray analysis shows that MMP-1 expression is dramatically up-regulated in a periostin-overexpressing HNSCC cell line as compared to the parent cell line (Figure 1 and Table 1) [10]. However, although these studies indicate the importance of MMP-1 expression in cancer malignancy, the detailed mechanism of MMP-1 in HNSCC progression is still unknown.

### 3.2. MMP-3 in HNSCCs

MMP-3/stromelysin-1 is highly expressed in the cancer microenvironment because MMP-3 is expressed by fibroblasts, endothelial cells and immune cells surrounding the tumor and cancer cells [37,38,39,40]. In breast cancer, the targeted overexpression of activated MMP-3 in the mammary gland induces the transition of normal cells to malignant epithelial cells, suggesting that MMP-3 promotes mammary carcinogenesis [41]. In addition, Kessenbrock *et al*. recently showed that the hemopexin domain of MMP-3 regulates the balance between canonical and noncanonical Wnt signaling in a nonproteolytic manner and affects adult epithelial stem cell function [42]. These findings suggest that MMP-3 potentially regulates cancer stem cells during tumor initiation and metastasis [42]. On the other hand, in HNSCCs, studies have shown that MMP-3 has a protective role in HNSCC tumorigenesis. McCawley *et al*. indicated that treatment of chemical carcinogens in MMP-3-deficient mice enhanced tumor growth as compared to control mice [43]. This report suggests that MMP-3 has an opposing role in cancer development and progression. However, gene expression profiling datasets show a significant increase in MMP-3 expression in HNSCC cases (Table 1, Table 2, Table 3, Table 4 and Table 5), suggesting that MMP-3 might have a role in HNSCC initiation or progression.

Markers of cancer stem cells, such as CD44 and glucose regulated protein 78 (GRP78), have been identified in HNSCCs, and cancer stem cells also contribute HNSCC tumorigenicity [44,45,46]. The molecular mechanisms of how MMP-3 affects HNSCC development and/or progression requires future investigation.

### 3.3. MMP-7 in HNSCCs

MMP-7/matrilysin is an enzyme that degrades collagens I, III, IV and V, fibronectin, vitronectin, laminin and elastin [47] and is expressed in variety of cancers including colon, prostate, esophageal and HNSCCs [48,49,50,51,52]. Gene expression analyses also show that MMP-7 is significantly overexpressed in almost all HNSCC datasets (Figure 2 and Table 1, Table 2, Table 3, Table 4 and Table 5). In fact, Chuang and colleagues have reported that active MMP-7 is associated with invasion in more invasive buccal squamous cell carcinoma [52]. Moreover, glutamate decarboxylase 1 (GAD1) is correlated with cellular invasiveness and migration by regulating β-catenin translocation and MMP-7 activation [53]. From these reports, although there is little doubt that the MMP-7 has an important role in HNSCC progression, its molecular mechanisms have not been identified.

A recent report by Dey *et al*. identified part of the mechanism of MMP-7 transcriptional regulation [54]. They clearly showed that high MMP-7-expressing breast cancer had relatively low phosphatase and tensin homolog (PTEN) levels both in clinical cases and cell lines, and the high levels of MMP-7 expression and enzymatic activity were abrogated by inhibition of phosphoinositide 3-kinase (PI3K), which is as main target of PTEN. Moreover, they also showed that higher levels of MMP-7 in triple-negative breast cancer patients were associated with poor outcome. Twenty-three percent of HNSCCs have PTEN mutations as determined by detailed exon sequencing [55,56,57]. These findings suggest that MMP-7 overexpression in many HNSCC cases may also be regulated by PTEN mutation.

### 3.4. MMP-10 in HNSCCs

MMP-10/stromelysin-2 constitutes a group with MMP-3/stromelysin-1. Although MMP-10 and MMP-3 are similar in their amino acid sequence and substrate specificity, expression patterns in normal and transformed cells are different [58,59,60,61].

According to gene expression profiling studies of MMPs, MMP-10 was highly expressed in the majority of HNSCC cases and could contribute to HNSCC progression (Table 1, Table 2, Table 3, Table 4 and Table 5 and Figure 2 and Figure 3). We have reported that MMP-10 plays an important role in the invasion and metastasis of HNSCC, and that invasion driven by MMP-10 is partially associated with p38 MAPK inhibition. Moreover, high expression of MMP-10 was frequently observed by immunohistochemistry and was significantly correlated with invasiveness and metastasis in HNSCC cases (116 cases) [12]. These observations are supported by other research groups showing that the PKCι-Par6α-Rac1 signaling axis promoted anchorage-independent growth and invasion of non-small-cell lung carcinoma (NSCLC) cells through the induction of MMP-10 expression [62]. In fact, MMP-10 expression is elevated in NSCLC tissues, and NSCLC patients whose tumors expressed high MMP-10 exhibited significantly worse survival than those whose tumors expressed low MMP-10 [62,63]. Tongue carcinoma cells treated with curcumin, a naturally occurring polyphenol derived from the root of *Curcuma longa*, inhibited cancer invasion through the suppression of MMP-10 expression [64]. Curcumin has been investigated as both a chemotherapeutic and chemopreventive agent through inhibition of NF-κB activation, leading to cell cycle arrest, apoptosis and suppression of proliferation. In many animal models of carcinogenesis, curcumin has achieved full therapeutic effects [65]. Indeed, in HNSCC, curcumin treatment resulted in suppression of cancer growth both *in vitro* and *in vivo* [66]. These reports suggest that tumor growth suppression by curcumin *in vivo* might also be involved in the reduction of MMP-10 expression. Further analyses are required in order to clarify the association of MMP-10 in cancer prevention and treatment by curcumin.

### 3.5. MMP-12 in HNSCCs

MMP-12 is an elastinolytic protease and degrades elastin, type IV collagen, fibronectin, laminin, vitronectin, entactin, heparin and chondroitin sulfate proteoglycans [67,68,69]. MMP-12 is mainly expressed by macrophages and can convert plasminogen into angiostatin. Thus, MMP-12 can effectively inhibit endothelial cell proliferation and angiogenesis, which limits tumor growth [70,71]. However, MMP-12 expression is actually increased in some cancer types including HNSCC and correlates with epithelial dedifferentiation and histological aggressiveness, suggesting that MMP-12 derived from cancer cells has a different role from that secreted by macrophages [13,14,72,73,74,75]. In fact, some studies have shown that the expression level of MMP-12 derived from cancer cells was higher in aggressive and poorly differentiated squamous cell carcinoma, while macrophage-derived MMP-12 was not abundant in early-stage cancer [72,76].

MMP-12 is up-regulated in HNSCCs by gene expression profiling (Table 1, Table 2, Table 3, Table 4 and Table 5 and Figure 2 and Figure 3) [13,14,72,73,74,76,77]. In addition, a recent report shows that MMP-12 expression correlates with extracapsular spread and nodal metastasis of HNSCCs [78]. From these findings, although MMP-12 is considered to be a marker for HNSCC aggressiveness, there are no studies showing whether MMP-12 inhibition can block cancer invasion and metastasis. 

### 3.6. MMP-13 in HNSCCs

Induction of angiogenesis is considered to be an important event in the metastatic cascade of tumors. It is widely accepted that tumor growth and metastasis are angiogenesis-dependent, and hence, blocking angiogenesis could be a strategy to arrest tumor growth [79]. Some MMPs, such as MMP-1, -2, -3, -7, -9, MT1-MMP and MT3-MMP, can contribute to distinct vascular events in tumors [80]. Among them, MMP-9, secreted by inflammatory cells, has a distinct role in tumor angiogenesis, by mainly regulating the bioavailability of vascular endothelial growth factor (VEGF). MMP-9 enables an angiogenic switch by increasing the bioavailability of sequestered VEGF for its receptor VEGFR-2 in pancreatic islet tumors [81]. In addition, the direct cleavage of matrix-bound VEGF by MMP-3, -7, -9 or MT3-MMP results in modified VEGF molecules with altered bioavailability, which changes the vascular patterning of tumors *in vivo* [82]. However, the degradation of ECM components and other extracellular molecules may generate fragments with new bioavailabilities that inhibit angiogenesis [83]. Thus, MMPs have dual functions as inhibiting and promoting angiogenesis, and the effects of MMPs on angiogenesis might be diverse.

MMP-13 is known as collagenase-3 and is active against a wide variety of ECM components [84]. Repairs of bone fractures in MMP-13-deficient mice are delayed, which suggests a critical role of MMP-13 in the process of angiogenesis during the healing of fractures [85]. Additionally, chicken MMP-13 is associated with cartilage and bone resorption and collagen remodeling in the angiogenic process [86]. Moreover, high expression of MMP-13 has been correlated to tumor behavior and prognosis [87]. Recently, MMP-13 produced from stromal fibroblasts promotes angiogenesis through increased protein levels of VEGF and VEGFR-2 in invasive areas of cancer [88].

In HNSCC cases, the datasets of gene expression profiling of MMPs show that MMP-13 expression was highly up-regulated (Table 1, Table 2, Table 3, Table 4 and Table 5 and Figure 1 and Figure 2). We have recently reported that MMP-13 was identified as a common up-regulated gene by HNSCC invasion-related factors and involved in tumor angiogenesis [89]. MMP-13 enhanced capillary tube formation both *in vitro* using human umbilical vein endothelial cells and *in vivo* using a rat aortic ring angiogenesis assay. Interestingly, apart from affecting the migration of endothelial cells, MMP-13 also promoted tumor angiogenesis through induction of VEGF-A secretion from fibroblasts and endothelial cells. In addition, MMP-13 expression was correlated with the number of blood vessels in HNSCC cases (64 cases). Indeed, high MMP-13 expression level is associated with aggressiveness of HNSCC [90]. Therefore, MMP-13 may have prognostic value in patient evaluation and is a potential therapeutic target for HNSCCs.

### 3.7. MMP-14 (MT1-MMP) in HNSCCs

Cancer cells form dynamic, actin-rich structures called invadopodia that localize to the ventral surface of cells and act as sites of matrix degradation via some types of enzymes including zinc-regulated metalloproteases [91,92,93,94,95]. MMPs are one of the major proteases that are present in invadopodia [96,97]. MMP-2, MMP-9 and MT1-MMP is associated with ECM degradation by invadopodia [98,99,100]. Loss of invadopodia formation in cancer cells by reduction of tyrosine kinase substrate with five SH3 domains (Tks5) abundance, a master regulator of invadopodia, reduced invasive behavior *in vitro* and tumorigenicity *in vivo* because cancer cells without invadopodia lack ECM degradative ability [101,102]. Furthermore, while it was previously believed that invadopodia are not required for cancer cell growth based on studies in monolayer cell culture, more recent studies reveal a role for invadopodia in growth in more physiological 3D ECM contexts [102]. Cancer cells with inhibition or knockout of MT1-MMP, a key proteinase localized at invadopodia structures, cannot grow in a 3D environment [103,104]. Some reports clearly show that the importance of invadopodia in HNSCC progression. Using a semi-orthotopic rat trachea model, overexpression of cortactin, one of the key components of invadopodia, promotes HNSCC growth *in vivo* through regulating an autocrine secretion of growth-promoting factors [105]. In addition, Clark *et al*. demonstrate that cortactin is an essential regulator of MMP secretion and ECM degradation at invadopodia in HNSCC. These data suggest that cortactin-induced MMP expression in invadopodia is a key event of HNSCC progression [99]. 

Although the results of gene expression profiling of MMPs (Table 1, Table 3, Table 5 and Figure 1, Figure 2 and Figure 3) and some other reports have shown that MT1-MMP expression increases in HNSCC cases and plays an important role in the formation of functional invadopodia during cancer progression [8,106,107,108,109], there are still significant technical problems that need to be solved for clinical assessment of cancer progression targeting invadopodia. The components of the invadopodia structure are also generally expressed in normal cells, and it is difficult to detect invadopodia as actin-rich structures in clinical specimens. Therefore, it may be required to identify a signature that is specifically expressed in invadopodia-forming cancer cells.

### 3.8. MMP-19 in HNSCCs

MMP-19, MMP-18, RASI-1, was cloned from a human liver cDNA library [110,111] and characterized as an enzyme that can cleave various ECM components, such as collagen IV, fibronectin and aggrecan [112,113]. Expression of MMP-19 is known in many cell types and its expression pattern is modulated according to the circumstances. Djonov *et al*. have reported that MMP-19 is strongly expressed in myoepithelial layer of the breast ductal tissue, benign tumor and their surrounding vasculature, but not in invasive tumor lesions [114]. In oropharyngeal squamous cell carcinoma, expression of MMP-19 is observed in healthy tissue areas, non-invasive tumor parts and also tumor invasive front, but its expression is absent in neoplastic regions [115]. Recent reports show that MMP-19 expression is increased in melanoma, ovarian cancer and glioma [116,117,118]. Müller *et al*. have shown that MMP-19 expression is up-regulated in melanoma vertical growth phase and metastases, and ectopic expression of MMP-19 facilitates transmigratory capacity through collagen type IV [116]. Triptolide, a compound extracted from the traditional chinese medicine preparation of *Tripterygium wilfordii* Hook F., can inhibit ovarian cancer invasion and tumor growth *in vivo* through transcriptional suppression of MMP-7 and MMP-19 [117]. Although expression of MMP-19 is up-regulated in some of the gene expression profiling (Table 1, Table 3 and Table 5), there is no report showing MMP-19 protein expression in HNSCC cases and experimental data that reveals role of MMP-19 in HNSCCs. Kudo *et al*. have demonstrated that highly invasive HNSCC cell line, MSCC-inv1, significantly overexpressed MMP-19 [4]. This suggests that MMP-19 may have a potential to facilitate HNSCC invasiveness. Further analyses are required to assess the role of MMP-19 in HNSCCs.

## 4. Conclusions

Gene expression profiling by microarray analysis indicates that many MMPs are highly expressed and contribute to tumor progression and poor outcome in various cancers including HNSCC. Among various cancers, HNSCC is well used for the study on MMPs. Here we introduced the roles of some MMPs, especially MMP-1, -3, -7, 10, -12, -13, -14 and -19 in HNSCC. Although the roles of MMPs in cancer behavior are evident, the agents targeting MMPs exhibited poor performance in clinical trials because of unknown side effects. MMPs are involved in not only ECM degradation for tumor dissemination, but also many important normal processes, such as cytokine activation and cleavage of cell-surface receptors. Thus, inhibiting MMPs could interfere with normal cellular behavior. Moreover, the spatio-temporal pattern of MMP expression both in normal and cancer tissues is complex and confusing. To reduce the unknown effects of MMP inhibitors, further analyses are needed to understand the entire aspect of MMP expression and modulation in all organs and cells.
